# Preventing preterm birth with progesterone: costs and effects of screening low risk women with a singleton pregnancy for short cervical length, the Triple P study

**DOI:** 10.1186/1471-2393-11-77

**Published:** 2011-10-24

**Authors:** Melanie A van Os, Jeanine A van der Ven, C Emily Kleinrouweler, Eva Pajkrt, Esteriek de Miranda, Aleid van Wassenaer, Martina Porath, Patrick M Bossuyt, Kitty WM Bloemenkamp, Christine Willekes, Mallory Woiski, Martijn A Oudijk, Katia M Bilardo, Marko J Sikkema, Johannes J Duvekot, Diederik Veersema, Jacqueline Laudy, Petra Kuiper, Christianne JM de Groot, Ben Willem J Mol, Monique C Haak

**Affiliations:** 1 Department of Obstetrics and Gynaecology, VU Medical Centre, Amsterdam, the Netherlands; 2 Department of Obstetrics and Gynaecology, Academic Medical Centre Amsterdam, the Netherlands; 3 Department of Neonatology, Emma Children's Hospital Academic Medical Centre, Amsterdam, the Netherlands; 4 Department of Obstetrics and Gynaecology, Máxima Medisch Centrum, Veldhoven, the Netherlands; 5 Department of Clinical Epidemiology, Academic Medical Centre, Amsterdam, the Netherlands; 6 Department of Obstetrics and Gynaecology, Leiden University Medical Centre, Leiden, the Netherlands; 7 Department of Obstetrics and Gynaecology, Academic Hospital, Maastricht, Maastricht, the Netherlands; 8 Department of Obstetrics and Gynaecology, Radboud University, Nijmegen, the Netherlands; 9 Department of Obstetrics and Gynaecology, University Medical Centre, Utrecht, the Netherlands; 10 Department of Obstetrics and Gynaecology, University Medical Centre, Groningen, the Netherlands; 11 Department of Obstetrics and Gynaecology, ZHT, Almelo, the Netherlands; 12 Department of Obstetrics and Gynaecology, Erasmus Medical Centre, Rotterdam, the Netherlands; 13 Diagnostiek voor u diagnostisch centrum, Eindhoven, the Netherlands; 14 Star Medisch Diagnostisch Centrum, Rotterdam, the Netherlands; 15 Ultrasound centre FARA, Ede, the Netherlands

## Abstract

**Background:**

Women with a short cervical length in mid-trimester pregnancy have a higher risk of preterm birth and therefore a higher rate of neonatal mortality and morbidity. Progesterone can potentially decrease the number of preterm births and lower neonatal mortality and morbidity. Previous studies showed good results of progesterone in women with either a history of preterm birth or a short cervix. However, it is unknown whether screening for a short cervix and subsequent treatment in mid trimester pregnancy is effective in low risk women.

**Methods/Design:**

We plan a combined screen and treat study among women with a singleton pregnancy without a previous preterm birth. In these women, we will measure cervical length at the standard anomaly scan performed between 18 and 22 weeks. Women with cervical length ≤ 30 mm at two independent measurements will be randomly allocated to receive either vaginal progesterone tablets or placebo between 22 and 34 weeks. The primary outcome of this trial is adverse neonatal condition, defined as a composite outcome of neonatal mortality and severe morbidity. Secondary outcomes are time to delivery, preterm birth rate before 32, 34 and 37 weeks, days of admission in neonatal intensive care unit, maternal morbidity, maternal admission days for preterm labour and costs. We will assess growth, physical condition and neurodevelopmental outcome of the children at two years of age.

**Discussion:**

This study will provide evidence for the usefulness and cost-effectiveness of screening for short cervical length at the 18-22 weeks and subsequent progesterone treatment among low risk women.

**Trial registration:**

Netherlands Trial Register (NTR): NTR207

## Background

Spontaneous preterm delivery is the single most important cause of perinatal mortality in the Western world [1]. It is known that cervical length measurement at 20 to 24 weeks gestation can identify women at increased risk for preterm delivery [2,3]. However, an effective treatment was not available until recently.

A breakthrough in the management of women at increased risk is the use of progesterone. Two randomized clinical trials demonstrated a reduction in preterm birth of 50% in women with a previous preterm birth [4,5]. The number of women who delivered prior to 32 weeks in both studies decreased from 20% to 10%. The effectiveness of progesterone was also addressed in a recent meta-analysis [6].

Relative to women allocated to placebo, those who received progestational agents (17[alpha]-hydroxyprogesterone caproate and other forms of progesterone) had lower rates of preterm delivery (26% versus 36%), corresponding to a number needed to treat to prevent one premature delivery of 10. In addition, women who had received progestational agents had lower rates of perinatal mortality (14.8% versus 17.1%).

The problem with the use of progesterone at present is that, based on current evidence, it can only be applied to women with a history of preterm birth. However, the large majority of spontaneous preterm delivery occurs in nulliparous women, and progesterone has not been assessed in those women [7]. On the other hand, prediction of preterm birth in nulliparous women has been studied widely. Fetal fibronectin, cervical length, upper genital tract infection, and bacterial vaginosis are predictors of preterm birth and in various combinations, proved to be stronger predictors, depending on race and gestational age. At 24 weeks, cervical length below 25 mm was the best predictor of spontaneous preterm birth at all gestational ages. Of the risk factors evaluated at 28 and 30 weeks, fetal fibronectin was the only significant predictor of spontaneous preterm birth < 32 weeks [8].

In a European cohort of 47.123 women, the cervix was 15 mm or less in 470. The proportion of preterm delivery in this group was almost 25%. In a randomised clinical trial attached to this study, the insertion of a Shirodkar suture in women with a short cervix did not substantially reduce the risk of early preterm delivery [9].

In view of the studies mentioned above, two conclusions can be drawn. First, progesterone is an effective treatment for women who are at increased risk for preterm birth due to a previous spontaneous preterm delivery. Second, routine sonographic measurement of cervical length at 22-24 weeks identifies a group of nulliparous women at increased risk of early preterm birth.

These two issues generate the hypothesis that the use of progesterone in women with a short cervical length can reduce the preterm delivery rate. In August 2007, the first randomised study on the effectiveness of progesterone in women with a singleton pregnancy and a short cervical length without a history of spontaneous preterm birth was published [4]. Spontaneous delivery before 34 weeks of gestation was less frequent in the progesterone group than in the placebo group (19% vs. 34% relative risk, 0.56). Progesterone was associated with a non-significant reduction in neonatal morbidity (8.1% vs. 13.8%; relative risk, 0.59, P = 0.17). There were no serious adverse events associated with the use of progesterone. The authors concluded that in women with a short cervix, treatment with progesterone reduces the rate of spontaneous early preterm delivery. However, as a significant effect on neonatal outcome was lacking, additional data are needed. We propose a national cohort study in which mid trimester cervical length is measured in 15.000 women, with subsequent randomisation of women with a decreased cervical length. The costs and effects of screening and treatment will be assessed, taking into account different cut-off values of short cervical length.

## Methods/Design

The study is set in the Dutch Obstetric Consortium a collaboration of obstetric practices in the Netherlands. Approximately 200 clinics, including university hospitals, teaching hospitals, non-teaching hospitals, ultrasound centres and midwifery practices will participate in this trial.

To conduct this trial we got approval from the Medical Ethical Committee, Academic Medical Centre, Amsterdam the Netherlands (MEC AMC 08-374 and 08-328). The study will be a cohort study with a randomised clinical trial embedded. We will screen a large cohort of women with low risk singleton pregnancies at 18-22 weeks gestation. Women with a short cervix will then be randomly allocated to receive either vaginal progesterone or placebo until 34 weeks.

### Procedures, recruitment, randomisation and collection of baseline data

In the Triple P *screening *study, we will offer low risk women with a singleton pregnancy the possibility to screen for a short cervix. Women < 18 years of age, women with a previous preterm birth < 34 weeks or symptoms of threatened preterm labour (uterine contractions, ruptured membranes), fetal congenital malformations and cervical or abdominal cerclage in situ will be excluded.

The cervical length is measured in addition to the standard anomaly scan at 18-22 weeks gestation, which all pregnant women are offered in the Netherlands. At the end of the standard anomaly scan, the measurement will be performed using a vaginal probe. The bladder of the participant needs to be empty. The measurement takes approximately three minutes and the shortest measurement is used. All participating sonographers will be asked to complete an e-learning module, specifically designed to learn the cervical length measurement technique. Subsequently, prior to participation in the study, each sonographer will be asked to obtain pictures of five measurements, which will be judged by the project team.

The cut off level for a short cervical length is ≤ 30 mm. Initially the cut of level was set at ≤ 25 mm. However, after we had screened 340 women, only 9 patients had a cervical length ≤ 25 mm at first measurement; a far lower rate than expected. Based on these findings the cut of level was altered to ≤ 30 mm, which corresponded to other recruiting trials.

When a cervical length ≤ 30 mm is measured, a second assessment is offered within two weeks.

This second measurement takes place in the same hospital. When the first measurement is done in an ultrasound centre, the second measurement takes place in the nearest hospital and is performed by a trained sonographer.

If at the second assessment the cervix remains ≤ 30 mm, women will be invited to participate in the randomised clinical trial. Women who are eligible for the study but do not give informed consent, are registered and data on their pregnancy outcome will be collected. Progesterone will not be offered to these women.

All eligible women with a singleton pregnancy and cervical length ≤ 30 mm will be informed shortly about the clinical trial by the supervising gynaecologist or midwife. Subsequently, a trained research nurse will inform the patient in detail. The patient will also receive written information about the study from the research nurse. Following the study information, the woman is given a minimal of five days time to consider participation in the clinical trial. In case of participation, informed consent is obtained for the treat study.

Once informed consent has been given, and patient data have been entered in a web based database, computerized randomisation will take place.

### Intervention

Each study participant will be given a blister pack labelled "Progesterone Study, "

that will contain either 200-mg capsules of micronized progesterone (Utrogestan, Besins International Belgium) or identical-appearing capsules of placebo (Medicaps).

Progesterone use did not show side effects in other studies, there was no difference observed between exposed and non exposed infants [10-12].

Study medication will be taken between 22 weeks and 34 weeks gestational age. The capsules will be self-administered vaginally by patients on a daily basis. The label codes indicating progesterone or placebo are only known in the central pharmacy. These data will be disclosed to the central office only after data on primary outcome have been collected. Researchers involved in the follow up program of the offspring of women participating in this study will remain blinded for a longer period. For emergency cases, a closed envelope with the label codes is available at the study centre. For purpose of the interim analysis the label codes will become available to the epidemiologist involved in the study as A and B. Baseline demographic, past obstetric and medical history will be recorded of all women who participate in the randomised study.

### Outcome measures

The primary outcome measure is composite poor neonatal outcome (death or severe morbidity). This composite morbidity rate contains the following variables: severe Respiratory Distress Syndrome (RDS), Bronchopulmonary Dysplasia (BPD), Periventricular Leucomalacia > grade 1, Intracerebral Haemorrhage > grade II, Necrotizing Enterocolitis (NEC) > stage 1, proven sepsis and death before discharge from the nursery. The primary outcome measure will be recorded 10 weeks after the expected term date.

Secondary outcome are time to delivery, preterm birth rate before 32, 34 and 37 weeks, days of admission in neonatal intensive care unit, maternal morbidity, maternal admission days for preterm labour and costs. Moreover, growth, physical condition including close examination of the genital tract, and neuro-developmental outcome of the child at 24 months (corrected) age will be assessed.

Next to secondary clinical outcome, the cost-effectiveness of screening for short cervical length (as done in Triple P *screening*), and subsequent treatment in case of a short cervix, will be assessed for several cut-off levels of cervical length.

### Follow up of women and infants

Follow up will be performed in all children. Questionnaires regarding child development will be sent to all participating families at the corrected child age of 24 months. Child outcomes to be measured are; length, weight and head circumference, medical history and medical consumption, physical examination with extra attention for the genital tract, neurological examination (including gross motor functional classification system for children with cerebral paresis). assessment of the cognitive and motor development, using the Bayley Scales of Infant Development, version III.

Child Behavioural Checklist, will be filled out by the parents to assess behavioural outcome. Ages and stages questionnaire will be filled out by all parents

### Statistical issues

#### Sample size calculation

We anticipated an adverse neonatal outcome in 14% of the pregnancies in which the cervical length is < 15 mm, and in 3% of the pregnancies with a cervical length between 15 mm and 30 mm. As we assumed that 1.7% of the pregnancies would show a cervical length < 15 mm, and 8.3% of the pregnancies a cervical length between 15 and 30 mm, the probability of bad neonatal outcome without intervention with progesterone in women with a cervix shorter or equal to 30 mm will be 5.0%. This percentage was expected to be reduced to 2.5% after use of progesterone. Assuming this decrease of the incidence of adverse neonatal outcome from 5% to 2.5%, using a two sided test with an alpha of 0.05 and a beta of 0.8, 1, 920 women (960 per arm) were needed in this study. As we expected that 10% of the women would have a cervix ≤ 30 mm, and with the assumption that 50% of the eligible women would participate, we need to screen almost 40, 000 women in the Triple P *screening *study. However since the start of the study, 3.000 women were screened resulting in only 70 patients with a cervical length ≤30 mm at the first measurement; a far lower rate of short cervix than we expected. Based on these findings, the feasibility of recruitment, the available budget and the example of similar international trials we have adjusted our protocol and plan to screen 15.000 women. Based on the current data, we hypothesize that we will be able to randomize 100 women. We aim to combine the trial data with that of other ongoing studies on the subject in an individual patient data meta analysis. (13).

### Data analysis

The results of the randomised clinical trial will be analysed according to the intention to treat principle. The effectiveness of progesterone versus placebo will be assessed by calculating relative risks and 95% confidence intervals. Time to delivery will be compared using Kaplan-Meier curves and log-rank tests. We will evaluate whether there is an interaction between the treatment effect and cervical length, and between treatment effect and ethnicity.

### Economic evaluation

#### General considerations

The economic analysis will be performed from the societal perspective. Both costs and outcomes will be discounted with a discount rate of 5%. The economic analysis of the trial itself is not of interest. If progesterone is found to decrease the probability of preterm delivery, then the savings due to decreased prematurity will always outweigh the costs of progesterone, which are negligible. The true economic question to be answered when the trial shows a beneficial effect is whether the costs of screening (number needed to screen to detect one woman with a short cervix) outweigh the cost reduction and health benefits from treatment with progesterone.

#### Cost analysis

The study design will enable us to compare the costs and effects of the following strategies:

I. no screening for cervical length

II. screening for cervical length, and treatment of women with a short cervical length

For each of these strategies, we will calculate the costs as well as the effects in terms of poor neonatal outcome (mortality and severe morbidity, see above). In the cost-effectiveness analysis, we will then calculate the costs per prevented case of bad neonatal outcome.

For strategy II, we will assess the screening strategy using different cut-off levels. We anticipate a stronger treatment effect in the group with a very short cervix, i.e. < 15 mm, whereas the treatment effect is likely to be less strong in the women with a cervix between 15 mm and 30 mm (number need to treat). On the other hand, the number of women with a short cervix will be smaller when a cut-off value of 15 mm is used than when the group with a cervix between 15 and 30 mm is considered to be abnormal (number needed to screen). Thus, the cost-effectiveness analysis will assess the balance between number needed to treat and number needed to screen.

For the cost analysis, the process of care is divided into three cost stages (antenatal stage, delivery/childbirth, postnatal stage) and three cost categories (direct medical costs [all costs in the health care sector], direct non-medical costs [costs outside the health care sector that are affected by health status or health care] and indirect costs of the pregnant woman and her partner [costs of sick leave]). Volumes of health care resource use are measured prospectively alongside the clinical study in all participating centres as part of the case report form.

Cost volumes in the antenatal stage consist of direct medical costs (e.g. prenatal checkups, costs of screening, admission due to threatened preterm birth, transport of patients to perinatal centres, and maternal monitoring [various lab tests; hospital care]). Costs during childbirth are dominated by the course of childbirth and type of delivery. Cost volumes in the postnatal stage consist of maternal care (hospitalisation etc.) and neonatal care (admission to neonatal intensive care/neonatology ward, outpatient visits) and primary care.

Health resource use outside the hospital is recorded by questionnaires. Direct medical volumes outside the hospital and direct non-medical volumes are valued using national reference prices. Indirect costs are quantified but remain unvalued. Study-specific costs are excluded from analysis.

## Discussion

Preterm birth is still a huge problem, with high mortality and morbidity rates. Progesterone is a safe and potentially effective treatment for prevention of preterm birth. Some studies have already proven the use of progesterone in high risk populations, showing that the number of preterm births can be reduced.

Unfortunately there is no evidence that progesterone use decreases neonatal morbidity and mortality. More research has to be done on the possible effects of progesterone on the neonatal morbidity and mortality rates.

Simultaneously with the Triple P trial, a number of other study groups in different countries have set up trials to evaluate the effect of progesterone on low risk pregnancy's to prevent preterm birth.

Recently one of those study's showed a reduction of preterm birth rate with the use of progesterone [13].

If the outcome and data of these studies are pooled a more conclusive statement can be made on this matter.

## Competing interests

The authors declare that they have no competing interests.

## Authors' contributions

BWJM, MCH, EP were involved in conception and design of the study. BWM, MCH and EP drafted the manuscript. All authors mentioned in the manuscript are members of the Triple P study project group; they participated in the design of the study during several meetings and two weekly conference calls. All authors mentioned in the manuscript are local investigators in the participating centers. All authors edited the manuscript and read and approved the final manuscript

## Pre-publication history

The pre-publication history for this paper can be accessed here:

http://www.biomedcentral.com/1471-2393/11/77/prepub
